# A Doctor in the House, An Ethical Consideration on Treating Their Family Members: A Mixed-Method Study

**DOI:** 10.7759/cureus.44230

**Published:** 2023-08-27

**Authors:** S Vijayalakshmi, S Ramkumar, T R Rajsri, S Prasanna, M Prince Rueban, U Pravin Anand, J Parasaran, S Kumar

**Affiliations:** 1 Community Medicine, Dhanalakshmi Srinivasan Medical College and Hospital, Perambalur, IND; 2 Community Medicine, Srilalithambigai Medical College, Chennai, IND

**Keywords:** doctors, public health, experience, ethics, treating family members

## Abstract

Background: The ethical dilemma of doctors treating their own family members has long been a contentious issue in the field of medicine. Despite these dilemmas, doctors may feel compelled to become involved in the care of family members and reluctant to set standards for themselves. Therefore, this study aimed to assess the experience of doctors in the treatment of their families in Perambalur District, Tamil Nadu, India.

Methodology: A mixed-method study was conducted among the doctors in Perambalur District, Tamil Nadu, India from December 2021 to October 2022. A semi-structured questionnaire was used to assess the socio-demographic details and the experience of doctors in treating their family members, followed by a focused group discussion (FGD). Data were analyzed using SPSS version 16 (SPSS Inc., Chicago). A scatter plot was created to visualize the relationship between age, experience of doctors, and confidence level with the frequency of treating family members. A chi-square test was performed to find any associations, and a p-value <0.05 was considered significant. For qualitative data, a fish herringbone model was constructed.

Results: A total of 72 doctors participated in the study. The study found that almost all the doctors (100%) received medical requests from family members, the median number of requests received in a year was 6.5 with an interquartile range of 4-8 and three-quarters (66.6%) of them accepted the requests and treated them. However, concerns about maintaining objectivity, emotional attachment, and loss of confidentiality were cited as primary reasons for not accepting all requests. The study also found a positive relationship between age, years of experience, and the frequency of treating family members. The FGD highlighted challenges related to potential risks in managing complex cases, emotional involvement impacting decision-making, conflicts of interest, and pressures from family members and societal norms.

Conclusion: In the present study, almost all the doctors received requests from their family members in the last year, and more than three-fourths of the doctors treated their family members. One-fourth of the doctors rejected requests from family members due to concerns about the potential loss of objectivity and the risk of misdiagnosing symptoms caused by emotional attachments. This study sheds light on the complexities and ethical considerations doctors face when treating family members. It emphasizes the need for medical ethics education in the curriculum to guide future doctors in making ethical decisions in such situations. Implementing clear-cut medical ethics guidelines in India would be instrumental in addressing these issues and ensuring ethical practices in the medical field.

## Introduction

The ethical dilemma of doctors treating their own family members has been a longstanding and contentious issue in the field of medicine. The complex interplay between professional responsibilities, patient welfare, and personal relationships gives rise to intricate ethical considerations that have intrigued the medical community for years. Doctors and other healthcare providers often find themselves navigating a delicate balance between their commitment to providing optimal care for patients and their innate desire to help their own loved ones [1].

While medical ethics guidelines and professional codes of conduct in various countries generally discourage doctors from treating family members, the topic remains an area of ongoing debate and reflection [2-5]. The challenges surrounding this issue are rooted in the potential for compromised objectivity, conflicts of interest, and the risk of emotional strain on the healthcare provider.

In the Indian context, the issue of doctors treating their family members is a complex ethical dilemma that is influenced by a range of cultural and social factors, i.e. in India, there is a strong emphasis on family values and the importance of caring for one's family members. In the medical field, this cultural value intersects with the notion of trust. Families tend to have implicit trust in their doctor's family members due to shared histories, experiences, and a perception of genuine care. This can lead to situations where doctors are not only responsible for their family's health but are also seen as the primary source of medical advice and treatment. This can create pressure on doctors to provide care for their family members, even if it creates a conflict of interest or goes against their professional obligations [6]. 

The Medical Council of India (MCI), which is the statutory body that regulates medical education and practice in India, has issued guidelines on the ethical conduct of medical professionals in India. In contrast to the other countries codes of ethics, Indian Medical Council (Professional Conduct, Etiquette and Ethics) Regulations, 2002 only states that “a physician can treat another physician and their immediate family members without seeking monetary compensation” and failed to lay a rule on health care professionals (HCPs) treatment with their family members [7-8].

Understanding the nuances of this ethical quandary is essential for promoting patient safety, maintaining professional integrity, and safeguarding the sanctity of the doctor-patient relationship. Over the years, a considerable body of research has emerged in developed countries to explore the various facets of this complex issue, shedding light on the decision-making processes and ethical reflections of healthcare professionals [1, 9-13].

When it comes to the ethical boundaries in the medical field, few topics stir up as much debate and controversy as doctors treating their own family members [9-11]. On one hand, the unique knowledge and expertise that doctors possess can be a valuable asset in providing specialized care for their loved ones [11]. On the other hand, potential conflicts of interest compromise objectivity, and blurred professional boundaries create a myriad of ethical concerns [10]. Should doctors be allowed to treat their family members, or does this practice violate the principles of fairness, impartiality, and patient autonomy? This delicate balance between familial duty and professional responsibility raises important questions about the moral obligations and ethical responsibilities of healthcare professionals.

Doctors treating their families are aspects of medicine that are not often discussed in the Indian Ethical Code (IEC). Despite these dilemmas, doctors may feel compelled to become involved in the care of family members and reluctant to set standards for themselves. There are vacuities in the literature in India regarding ethical concerns raised in treating their family members from doctors' perspectives. So, this study aimed to assess the experience of doctors in the treatment of their families in Perambalur District, Tamil Nadu, India.

## Materials and methods

Study design

A mixed-method explanatory sequential study [cross-sectional survey followed by a focused group discussion (FGD)] was conducted in the Perambalur district of Tamil Nadu, India.

Study period and participants

The study was conducted from December 2021 to October 2022, spanning a period of 11 months. The complete list of doctors registered in the Indian Medical Association's local chapter was obtained. Invitations were sent to the doctors practicing in Perambalur to participate in the study. All willing doctors who provided consent to participate and were practicing in the Perambalur district, Tamil Nadu, were included in the study.

Study tool

A predesigned and pre-tested questionnaire was used to collect socio-demographic data, as well as information on the doctors' treating their family members. The questionnaire collects socio-demographic data to gather information about the participants' background characteristics. This includes items such as age, gender, educational qualifications, professional experience, specialty or field of practice, and employment setting. Another section of the questionnaire focused on the experiences of doctors in providing healthcare to their family members. It explores aspects, such as the frequency of providing care, types of medical services provided, challenges faced in treating family members, emotional or ethical considerations, and the impact on their personal relationships. Following the quantitative study, FGD was conducted among the doctors. A total of four rounds were held to elicit the various factors that influence treating family members.

Data collection procedure

Quantitative Data Collection - Cross-Sectional Survey

All willing doctors practicing in the Perambalur district were included in the study. The total number of participants was 72 out of 75 approached, resulting in a 96% response rate. After obtaining informed consent, a direct interview was conducted by the principal investigator using a predesigned and pre-tested questionnaire. Information regarding each doctor's socio-demographic data and information related to doctors treating their family members, as per the study tool, was collected.

Qualitative Data Collection - Focused Group Discussion (FGD)

Doctors who participated in the cross-sectional survey were invited to take part in the FGDs. A total of four FGDs were conducted to elicit various factors influencing doctors when treating their family members. In each FGD, around six to eight doctors participated. The discussion continued until the saturation of ideas was reached. The discussions during the FGDs were recorded and transcribed for further analysis.

Statistical analysis

Quantitative study data were entered into a Microsoft Excel spreadsheet and analyzed using SPSS version 18 (SPSS Inc., Chicago). Descriptive statistics, such as percentages, rates, and ratios, were calculated. Normality of the data was assessed using SPSS version 18, and non-parametric tests were adopted. The chi-square test was performed to assess associations among categorical variables, with a p-value less than 0.05 considered statistically significant. Spearman correlation analysis was used to determine the correlation between doctors' age, years of experience, and confidence level, with the frequency of treating family members. A scatter plot was generated to visually represent the correlation.

Thematic analysis was conducted on the data gathered from the FGDs [14]. After collecting the FGD data, a thorough reading was undertaken to gain a comprehensive understanding of the content. Subsequently, the initial coding process began. Relevant excerpts from the doctors' discussions regarding challenges in treating family members were extracted and labeled with descriptive codes. These codes encapsulated the core content of each piece of data. Themes were derived from the initial codes, grouping together similar codes that revolved around common ideas. This process led to the identification of several overarching themes that captured the various dimensions of challenges faced by doctors in such situations. The identified themes were challenges in patient outcomes, professional relationships, and ethical and practical considerations. 

Further coding was carried out within each theme. The responses provided by the doctors were organized using a framework, the "fish herringbone model." This model allowed for the systematic arrangement of responses, making it easier to visualize and analyze the connections and relationships among different themes and sub-codes.

Ethical considerations

Written informed consent was obtained from all doctors who willingly participated in the study before the commencement of the research. The anonymity of the participants was rigorously upheld throughout the entire study duration to ensure the utmost confidentiality of their identities and responses. In order to maintain ethical integrity, the study received ethical clearance from the Institute Ethical Committee, affirming that the research was conducted in accordance with established ethical standards and guidelines.

## Results

Of the 75 doctors approached, 72 (96%) participated in the survey. Table 1 displays the general characteristics of the doctors, including their median age [interquartile range (IQR)] of 35 (30-44.5) and three-quarters of them being male. Approximately 68.1% of the participants practiced in urban areas, and the median years of experience were 6 (IQR: 4-8.7) years.

**Table 1:** General characteristics of the doctors (n=72).

Characteristics		Frequency
Age in years	Median (IQR)	35 (30-44.5)
Gender n(%)	Male	54 (75.0)
	Female	18 (25.0)
Qualification n(%)	MBBS	18 (25.0)
	MD/ MS	54 (75.0)
Place n(%)	Urban	50 (69.4)
	Rural	22 (30.6)
Years of experience	Median (IQR)	06 (4-8.7)

The data presented in Table 2 shows the experience of doctors in treating their family members. The median number of requests received in a year was 6.5 with an interquartile range of 4-8. Among the participants, 72.2% received five or more requests in a year. The type of requests varied, with most participants receiving requests for advice (95.8%) and consultations (83.1%). Fewer participants received requests for second opinions (35.5%) and other types of requests (9.7%).

**Table 2:** Characteristics of doctors’ experience in treating their family members. *Multiple response IQR, inter quartile range

Characteristics		Frequency
Frequency of medical requests received from family members	Median (IQR)	6.5 (4-8)
Number of requests received in a year n(%)	<5	20 (27.8)
	≥5	52 (72.2)
Type of requests* n(%)	-Asked advice	69 (95.8)
	-Consultations	60 (83.1)
	-Second opinion	22 (35.5)
	-Others	6 (9.7)
Accepted all the requests and treated n(%)	-Yes	48 (66.6)
	-No	24 (33.4)
Place of treatment given to family members n(%)	Home	31 (64.5)
	Clinics/ Tertiary hospital	17 (35.5)
Have you preferred second opinion while treating a family member? (n=48) n(%)	-Yes	17 (54.8)
	-No	31 (45.2)
Confidence level of doctors for treating the family members n(%)	Not at all satisfied	5 (6.9)
	Slightly satisfied	13 (18.1)
	Moderately satisfied	14 (19.4)
	Very satisfied	22 (30.6)
	Completely satisfied	18 (25)

Regarding the acceptance of requests for consultations, 66.6% of participants reported accepting all requests and treating their family members, while 33.4% reported not accepting some requests. The participants' confidence levels for treating their family members among doctors varied, with 30.6% of participants reporting being very satisfied with their performance, followed by moderately satisfied (19.4%), completely satisfied (25%), slightly satisfied (18.1%), and not at all satisfied (6.9%) (Table 2).

**Figure 1:**
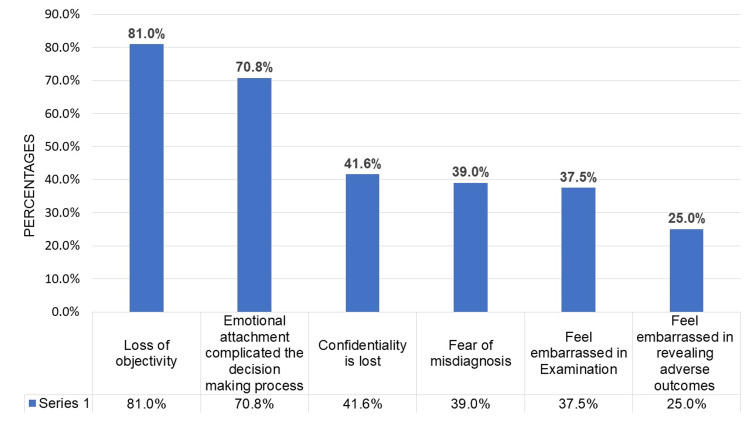
Reasons for not accepting all the requests from family members (n=24).

Figure 1 shows the multiple-response bar chart to assess the reasons for not accepting all requests from family members. The majority of respondents (81.0%) cited concerns about maintaining objectivity as the primary reason for not accepting all requests. Additionally, 70.8% of them reported that emotional attachment complicated the decision-making process, while 40% reported a loss of confidentiality when treating their family members.

**Figure 2:**
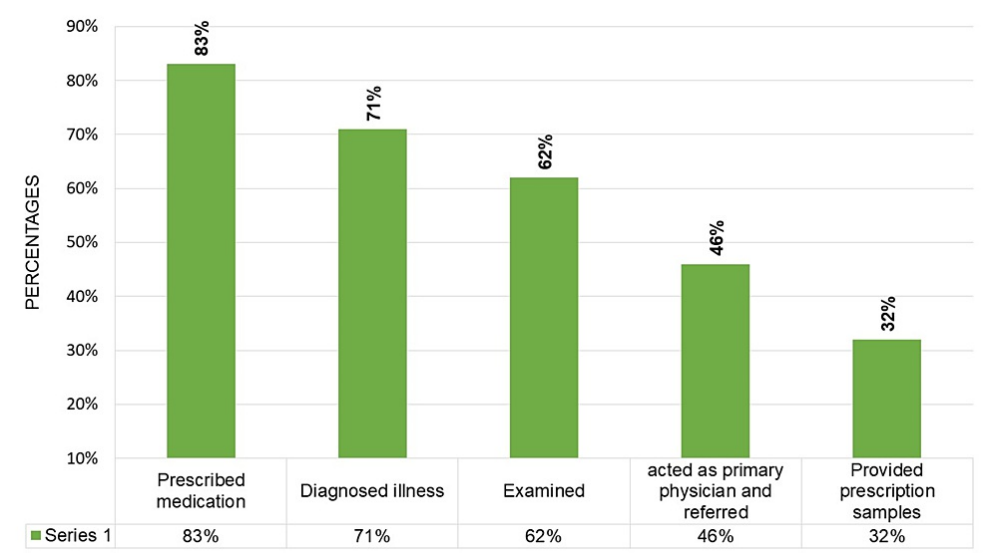
Type of services provided by doctors for their family members (n=48).

Figure 2 shows the multiple response bar chart to assess the type of services provided by the doctors to their family members. The study found that doctors who treat their family members provide a variety of services, including providing prescription samples (20%), acting as a primary physician, referring their family member to a specialist if needed (46%), diagnosing their illness (62%), examining them (62%), and prescribing medication (83%).

Table 3 shows an association between treating family members and doctors. A significant association was found between residence and treating family members with a p-value <0.05. There were no significant associations found between gender, and qualification with treating family members with a p-value >0.05.

**Table 3:** Association between treating family members and doctors profile (n=72). p-value < 0.05 - significant

Characteristics		Treating family members		p-Value
		Yes n (%)	No n (%)	
Gender	Male	37 (68.5)	17 (31.5)	0.546
	Female	11 (61.1)	07 (38.9)	
Qualification	MBBS	09 (50)	09 (50)	0.083
	MD/MS	39 (72.2)	15 (27.8)	
Residence	Rural	38 (76.0)	12 (24.0)	0.011
	Urban	10 (45.5)	12 (54.5)	

**Figure 3:**
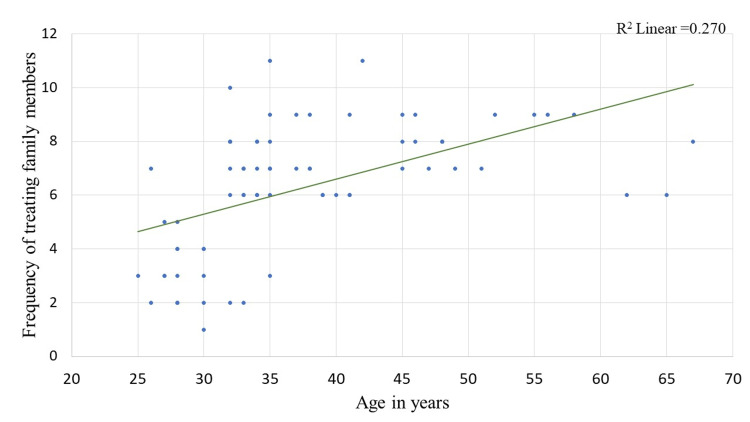
Scatter plot showing the correlation between age of doctors and the frequency of treating family members. Correlation co-efficient value for age (0.619; p-value < 0.001)

**Figure 4:**
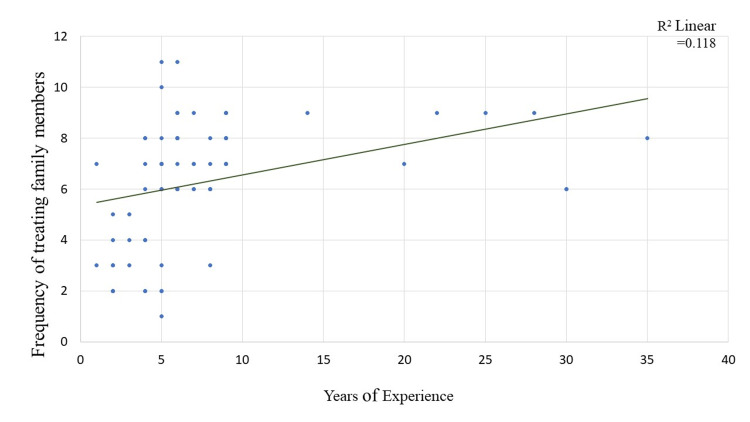
Scatter plot showing a correlation between years of experience with treating the family members. Correlation co-efficient value for years of experience (0.547; p-value < 0.001)

**Figure 5:**
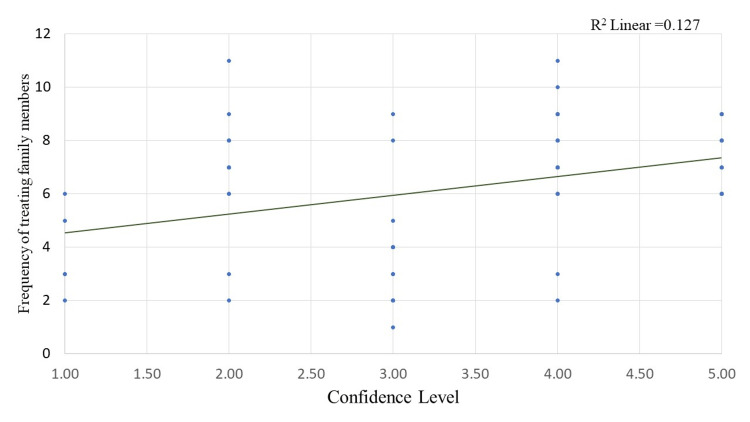
Scatter plot showing the correlation between the confidence level of doctors and the frequency of treating family members. Correlation co-efficient value for confidence level (0.341; p-value = 0.003)

Scatter plots in Figures 3-5 show the relationship between the age, years of experience of doctors and confidence level with the frequency of treating family members. The correlation coefficient value for age and frequency of treating family members is 0.619, indicating a positive correlation. Similarly, the correlation coefficient value for years of experience and confidence level with the frequency of treating family members is 0.547 and 0.341, respectively, also indicating a positive correlation, i.e., the study found that one unit increase in age, years of experience, and confidence level of doctors results in 0.619 times, 0.547 times, and 0.341 times increase in the frequency of treating the family members respectively. The plot also shows a significant p-value of less than 0.05, indicating that this relationship is not due to chance and is likely to be a true association.

 

**Figure 6:**
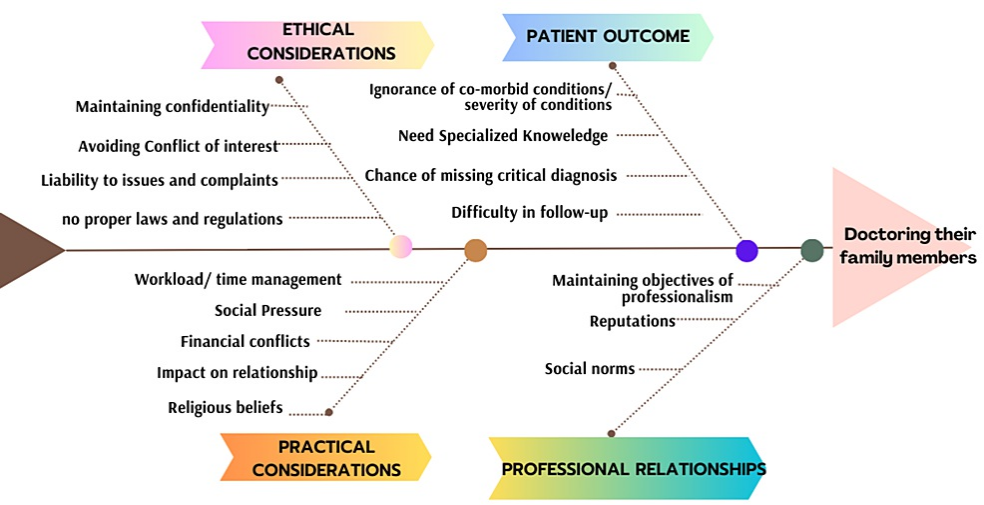
Fish Herringbone model for the factors affecting doctors treating their family members.

From the results of FGD, the fish herringbone model was constructed. The central issue in the model being examined is doctors treating their family members, and the main categories of factors that can affect this decision are patient outcome, professional integrity, and ethical and practical considerations as shown in Figure 6. 

## Discussion

In the present study, the aim was to assess the experiences of doctors when providing medical care to their family members in the Perambalur district, Tamil Nadu. Notably, the study found substantial participation from male participants, comprising 75% of the total sample, which aligns with the findings of similar studies conducted by Knuth et al. [9], Krall et al., [10] La Puma et al., [11] Giroldi et al., [15] and Matsunga et al. [16]. 

In this study, the median age of the participants was 35 years, with an age range of 30-44.5 years. This finding is consistent with the median age group of doctors reported by Knuth et al. and La Puma et al., where it ranged from 32 to 35 years [9, 11]. However, Giroldi et al.’s and Matsunga et al.’s studies reported participants aged over 45 years [15-16], which indicates a difference in age groups between the studies. The variation in the age groups across studies may be attributed to the different study settings and the specific populations being investigated. Factors such as the healthcare system in the region, availability of medical services, and characteristics of the surveyed HCPs might contribute to the gender and age distribution observed in each study.

Regarding the educational background of the doctors, a majority of the participants had completed MD/MS (Doctor of Medicine/Master of Surgery), indicating a higher proportion of specialists among the respondents. The median years of experience for the doctors were 6 years, with a range of 4-8.7 years. These findings were similar to La Puma et al., Matsunaga et al., and Mucke et al., where the years of experience ranged from 4 to 10 years [11, 15-16]. The findings of our study suggest that the study area predominantly had relatively young doctors who were actively engaged in clinical practice.

Treating their family members is another paramount concern among doctors. In the current study, nearly all the doctors received medical requests from their family members in the past year, and of these requests, 66.6% were accepted and treated by the doctors. The median frequency of medical requests received from family members was 6.5 (4-8) times. Approximately 72% of them received medical requests more than five times a year. Similar findings were observed in the studies conducted by Knuth et al., Puma et al., Matsunaga et al., and Mucke et al., where more than 90% of doctors received requests from their family members, and the acceptance rates ranged from 60% to 78% [9, 11, 16-17]. This suggests that doctors, irrespective of the area, might experience a relatively high frequency of medical requests from their family members, which could add to their workload and responsibilities.

In the current study, after accepting the request, 83% of the doctors administered medication, 71% diagnosed illnesses, and 62% conducted physical examinations. The findings were similar to the studies conducted by Giroldi et al. and Matsunaga et al. [15-16]. These studies reported that the majority of the family members' requests were treated with medications, and only less than one-third of them were examined before receiving prescribed medicine. This shows that when doctors accept medical requests from their family members, they tend to rely heavily on medication as a treatment approach. While a significant proportion of them diagnose illnesses before treatment, the percentage of physical examinations conducted is relatively lower. This could indicate that healthcare professionals' familiarity with their family members' medical history and symptoms might influence their treatment decisions. If doctors treat the disease inadequately without proper examination and investigations, there may be a chance of getting a relapse or deterioration of the patient’s condition, which would be a serious ethical concern to be tackled [11-12]. Addressing these ethical concerns was very less in India compared to other developed countries [1-5, 7-8].

In the present study, approximately 26% of the doctors did not accept treatment requests from their family members. The majority of respondents (81.0%) cited concerns about maintaining objectivity as the primary reason for not accepting all family members' requests. Additionally, 70.8% of them reported that emotional attachment complicates the decision-making process, and 40% of respondents mentioned concerns about the loss of confidentiality when treating their family members. The findings were consistent with La Puma et al., Latessa et al., and Anyanwu et al. [11-13]. These findings suggest that doctors aim to avoid biases or favoritism that may arise from emotional connections with their family members. Moreover, they feel that emotional bonds could interfere with their ability to make unbiased decisions and may result in hesitation when disclosing sensitive information to their family members. This raises the question of whether emotional attachments and the fear of jeopardizing relationships with loved ones may potentially impact the doctors' skills, even within their field of expertise. Similarly, the ethics codes of the UK, USA, and Canada now prohibit doctors from operating on their family members [2-5]. However, in India, there is no written rule authenticating that doctors should not treat their family members [7-8]. 

Assessing the confidence level of doctors in treating their family members is indeed essential to mitigate the risks of misdiagnosis and ensure the provision of quality healthcare. In the present study, it was observed that nearly half of the doctors reported feeling confident in treating their family members. It is important to note that a sense of confidence is crucial for any healthcare provider, but it is also essential to recognize that treating family members presents unique challenges.

On examining the association, in the present study, we found that residence plays a vital role in providing treatment for family members. Additionally, a positive linear relationship was found between age, years of experience, and confidence level with the frequency of treating family members. The study suggests that the location or residence of doctors might influence their willingness or ability to provide medical treatment to their family members. This could be due to various factors, such as the availability of medical facilities, work-life balance, or cultural norms in different areas. Further research might be needed to explore the specific reasons behind this association.

The positive linear relationship between age and the frequency of treating family members indicates that older doctors tend to treat their family members more frequently than younger doctors. This could be attributed to the accumulation of experience and expertise over time, leading to higher confidence in providing medical care to their loved ones.

The study also found a positive linear relationship between the number of years of experience and the frequency of treating family members. This suggests that as doctors gain more experience in their field, they may become more comfortable and adept at treating their family members, which could lead to an increase in the frequency of providing medical care.

In the Fish Herringbone model, each factor is connected to the central issue, indicating its relationship and influence on doctors treating their family members. It highlights the complexity and multifaceted nature of the issue, and how various factors interact and impact the final decision-making process. The Fish Herringbone model helps visualize the interconnections and facilitates a comprehensive understanding of the factors affecting doctors' decisions when providing medical care to their family members.

During the FGD, most of HCPs highlighted that treating family members might lead to overlooking or underestimating the presence or severity of other health conditions the patient might have, as family members may not reveal the complete medical history, and also, some medical conditions might require expertise beyond the scope of the treating doctor, leading to potential risks in managing complex cases. Doctors stated that emotional attachment may lead to overlooking certain symptoms or delaying the diagnosis, affecting patient outcomes. They stressed that ensuring consistent follow-up might be challenging due to personal and professional responsibilities, which can impact the patient's ongoing care and recovery.

Regarding ethical considerations, doctors highlighted that emotional involvement might compromise their objectivity in decision-making and treatment. Also, treating family members may create conflicts of interest between professional duties and personal relationships. They highlighted that legal and ethical liabilities may arise if there are unfavorable outcomes or allegations of malpractice, and the absence of clear guidelines might lead to uncertainty in navigating ethical challenges.

Treating family members might add to the doctor's workload and affect their ability to manage other patients effectively. Furthermore, family members' expectations and societal norms may put pressure on doctors to provide care, irrespective of professional considerations, and financial implications or expectations from family members may affect treatment decisions. The findings of FGD are highlighted through the Fish Herringbone model to picture the understanding of the complexities of doctors treating their family members. It sheds light on the challenges and ethical considerations healthcare professionals face in such situations, and it recommends addressing these issues through awareness and protocol development. 

Though the ethical considerations surrounding doctors treating their family members are of paramount importance in today's context. It is crucial to approach the interpretation of the study findings with careful consideration. While the positive associations between age, years of experience, and the frequency of treating family members do indicate trends within the study population, it is imperative to recognize that individual variations and other factors might also impact doctors' choices to administer medical care to their family members. Furthermore, it is worth noting that this study may be subject to certain limitations, such as sample size and study design, which could potentially affect the applicability of the results. The overrepresentation of male participants could potentially introduce gender bias into the study's conclusions. Gender-related elements, including communication patterns, attitudes, and experiences, could potentially be influenced by the gender composition of the sample. This, in turn, might lead to findings that are skewed toward reflecting male perspectives.

Furthermore, reforming the MBBS curriculum to address the ethics of treating family members is a pivotal step in fostering a healthcare workforce that embodies compassion, ethical awareness, and competence. By instilling these values during their education, medical students can become exemplary professionals who prioritize patient’s well-being, uphold ethical principles, and contribute positively to the medical profession and society as a whole.

## Conclusions

In the present study, almost all the doctors received requests from their family members in the last year, and more than three-fourths of the doctors treated their family members, which raises ethical concerns. The major services provided were prescribing medication and diagnosing illnesses, rather than conducting thorough examinations of the patients. One-fourth of the doctors rejected requests from family members due to concerns about the potential loss of objectivity and the risk of misdiagnosing symptoms caused by emotional attachments. An association was found between residence and family requests, indicating that rural doctors receive more requests than urban ones. Age, experience, and confidence level positively correlated with the increased frequency of requests from family members. On FGD, doctors stated that while treating family members, factors like patient outcomes, ethical considerations, professional responsibilities, and personal considerations might be looked upon.

Being a doctor treating family members can be challenging, and relevant issues should be considered before taking on such a role. Doctors should receive targeted training on managing the challenges associated with treating family members. This training could cover effective communication strategies, ethical decision-making frameworks, and guidelines to mitigate potential conflicts of interest. Creating a supportive environment for doctors to openly discuss these challenges within their medical teams could also foster better decision-making. Conducting a more comprehensive study with a balanced representation of both male and female participants could yield deeper insights into gender-specific challenges in treating family members. Exploring the impact of cultural and societal norms on doctors' decisions in different regions could also contribute valuable cross-cultural perspectives. Future studies could investigate patient perspectives on being treated by family member doctors. This could provide insights into patient preferences, potential benefits, and challenges, guiding the development of patient-centered protocols.

It is time that medical colleges incorporate curricula addressing these issues to prepare future doctors on how to decide whether to treat ailing family members in their time of need. Research tracking the long-term effects of doctors treating family members could shed light on the impact of these decisions on professional relationships, patient outcomes, and the doctors' well-being over time. Further research is needed to establish clear-cut medical ethics in our country and address these issues.

## References

[1] Chen FM, Feudtner C, Rhodes LA (2001). Role conflicts of physicians and their family members: rules but no rulebook. West J Med.

[2] (2023). General Medical Council. "Good Medical Practice". GMC, UK. 25 Mar.

[3] (2013). American Medical Association. Ethics Opinion 8.19 - Self-treatment or treatment of immediate family members. https://code-medical-ethics.ama-assn.org/ethics-opinions/treating-self-or-family#:~:text=In%20general%2C%20physicians%20should%20not,no%20other%20qualified%20physician%20available.

[4] American College of Physicians (1999). Internal medicine-ACP internist. Should doctors treat their relatives?. ACP Internist. Ethics case study, ACP observer.

[5] (2023). The College of Physicians and Surgeons of Ontario (2007). Treating self and family members. http://pubs.sciepub.com/ajphr/2/3/6/index.html.

[6] Gautam S, Jain N (2010). Indian culture and psychiatry. Indian J Psychiatry.

[7] Medical Council of India (2023). Indian Medical Council (Professional Conduct, Etiquette and Ethics) Regulations. Indian Medical Council (Professional Conduct, Etiquette and Ethics) Regulations.

[8] (2023). Medical Council of India. The Indian Medical Council (Amendment) Act. https://www.nmc.org.in/documents/e_Gazette_Amendments/IMC%20Amendment%20Act-2016-05.08.2016.pdf.

[9] Knuth J, Bulian DR, Ansorg J (2017). When you operate on friends and relatives: results of a survey among surgeons. Med Princ Pract.

[10] Krall EJ (2008). Doctors who doctor self, family, and colleagues. Wisconsin Med J.

[11] La Puma J, Priest ER (1992). Is there a doctor in the house? An analysis of the practice of physicians’ treating their own families. JAMA.

[12] Latessa R, Ray L (2005). Should you treat yourself, family or friends?. Fam Pract Manag.

[13] Anyanwu EB, Abedi HO, Onohwakpor EA (2014). Ethical issues in treating self and family members. Am J Public Health Res.

[14] Braun V, Clarke V (2006). Using thematic analysis in psychology. Qualitat Res Psychol.

[15] Giroldi E, Freeth R, Hanssen M (2018). Family physicians managing medical requests from family and friends. Ann Fam Med.

[16] Matsunaga T, Kaneko M, Fetters MD (2022). Japanese primary care physicians’ experience in treating their family members: a cross-sectional study. Ann Fam Med.

[17] Mücke NA, Schmidt A, Kersting C (2022). General practitioners treating their own family members: a cross-sectional survey in Germany. BMC Prim Care.

